# Supplementary material for: Antibody Fab‐Fc properties outperform titer in predictive models of SIV vaccine‐induced protection
Source: Mol Syst Biol. 2019 May 2;15(5):e8747. doi: 10.15252/msb.20188747 (PMC6497031; doi:10.15252/msb.20188747)
Supplement: Supplementary file 1 — Appendix [file MSB-15-e8747-s001.docx]

**Appendix**

**Antibody Fab-Fc Properties Outperform Titer in Predictive Models of SIV Vaccine-Induced Protection**

Srivamshi Pittala^1^, Kenneth Bagley^2^, Jennifer A. Schwartz^2^, Eric P. Brown^3^, Joshua A. Weiner^3^, Ilia J. Prado^2^, Wenlei Zhang^2^, Rong Xu^2^, Ayuko Ota-Setlik^2^, Ranajit Pal^4^, Xiaoying Shen^5^, Charles Beck^6^, Guido Ferrari^6^, George K. Lewis^7^, Celia C. LaBranche^6^, David C. Montefiori^8^, Georgia D. Tomaras^5^, Galit Alter^9,10^, Mario Roederer^11^, Timothy R. Fouts^2,12^, Margaret E. Ackerman^3^, Chris Bailey-Kellogg^1*^.

|  | **Table of Contents** |
| --- | --- |
| **1** | **Appendix Figure S1** |
| **2** | **Appendix Figure S2** |
| **3** | **Appendix Figure S3** |
| **4** | **Appendix Figure S4** |
| **5** | **Appendix Figure S5** |
| **6** | **Appendix Figure S6** |
| **7** | **Appendix Figure S7** |

**Appendix Figure S1.** The antigens **(A)** and detection reagents **(B)** used for generation of high-throughput multiplexed data are shown. Heat maps of data for Fc Array features **(C)** and titer features **(D)** are shown. Columns represent Fab:Fc measurements and rows represent animals. Each feature was normalized to zero mean and unit variance, and truncated to 3 standard deviations from its respective mean.

**Appendix Figure S2.** **(A)** Overview of the predictive analysis framework. Cross-validation is performed using the input measurements, training the models on a subset of the samples and making predictions on the remaining samples. Cross-validation is repeated with different splits of training and testing sets and prediction performance is measured for each repetition. To visualize a subset of features with strongest correlations to the target labels, a final model is trained using all samples of the data and modeling parameters that provide best cross-validation performance. For permutation testing, the target labels are shuffled across samples and cross-validation is performed. This shuffling of labels and cross-validation is repeated, and prediction performance is measured. The prediction performance of models attained with actual data is compared to that of models with permuted data. **(B)** Overview of the antigen-specific titer adjustment procedure. The component of antigen-specific titer for each Fc Array measurement corresponding to the antigen is computed by scaling the titer (anti_IgG) by a factor equal to the Pearson correlation coefficient between the two measurements. The resulting measurements (titer-independent components) after subtracting the titer components from the Fc Array measurements have zero correlation with the titer vector. This procedure is performed independently for each antigen specificity used in the Fc Array.

**Appendix Figure S3. Group-wise analysis of features contributing to the final survival model.** For each feature, the values within each group are tested for significant differences from the values within the other three groups combined (Wilcoxon-Mann-Whitney).

**Appendix Figure S4. Comparison of prediction performance of survival models using different subsets of Fc array measurements.** Actual represents the case with all 82 measurements and No Gag, No Pol, and No Env represent cases with Fc Array measurements corresponding to the respective antigens discarded. The p-values represent the tail probabilities of the mean C-index of the actual models in each of the three distributions.

**Appendix Figure S5. Combination of predicted risk with previously employed measures of immune balance. (A)** A high T-cell response (IFNγ above 75^th^ percentile, y-axis) was previously found to exacerbate risk (Fouts *et al*, 2015), and here explains three animals (in red circles) predicted to be of low risk while actually remaining uninfected for only a small number of challenges (x-axis). **(B)** KM curves for animals partitioned based on the combination of predicted risk score and IFNγ response, mirroring the previous partitions based on ADCC and IFNγ (Fouts *et al*, 2015) to show the importance of a balance between humoral (here risk score) and cellular (IFNγ) responses. **(C)** A robust humoral response (ADCC above 0, x-axis; animals at 0 are stacked up below 0 for visualization purposes) was previously found to mitigate risk (Fouts *et al*, 2015), but here does not explain any of the animals misestimated to be of low risk while remaining uninfected for a small number of challenges (x-axis).

**Appendix Figure S6. Visualization of the two Fc Array features that were identified by the logistic classifier as correlates of IL-12 adjuvanted group, but did not show correlation with protection.** The IL-12 group animals exhibited significantly higher response compared to the Empty and LTA1 groups (left panels), but no strong correlation with protection (right panels).

**Appendix Figure S7. Four-way adjuvant group classification. (A)** Confusion matrix of test set predictions from the representative 8-fold cross-validation using multinomial logistic regression. The reverse diagonal shows the proportion of true positive predictions for each adjuvant group. **(B)** Log odds of predicting the true class label for each animal in the four adjuvant groups. Each animal is colored by its predicted group’s color. **(C)** Comparison of classifier’s performance from repeated cross-validation using actual and permuted data (Tail probability). **(D)** Logistic regression coefficients for the features selected by the final model trained using all samples. The bar for each feature represents the coefficient value for each of the four adjuvant groups (represented by their respective color). **(E & F)** Bi-plot visualization of the top two coefficients by magnitude for (E) Empty and (F) IL-12 adjuvanted groups.
